# Tailoring Eigenmodes at Spectral Singularities in Graphene-based PT Systems

**DOI:** 10.1038/s41598-017-11231-y

**Published:** 2017-09-12

**Authors:** Weixuan Zhang, Tong Wu, Xiangdong Zhang

**Affiliations:** Beijing Key Laboratory of Nanophotonics & Ultrafine Optoelectronic Systems, School of Physics, Beijing Institute of Technology, Beijing, 100081 China

## Abstract

The spectral singularity existing in PT-synthetic plasmonic system has been widely investigated. Only lasing-mode can be excited resulting from the passive characteristic of metallic materials. Here, we investigated the spectral singularity in the hybrid structure composed of the photoexcited graphene and one-dimensional PT-diffractive grating. In this system, both lasing- and absorption-modes can be excited with the surface conductivity of photoexcited graphene being loss and gain, respectively. Remarkably, the spectral singularity will disappear with the optically pumped graphene to be lossless. In particular, we find that spectral singularities can exhibit symmetry-modes, when the loss and gain of the grating is unbalanced. Meanwhile, by tuning the loss (gain) of graphene and non-PT diffraction grating, lasing- and absorption-modes can also be excited. We hope that tunable optical modes at spectral singularities can have some applications in designing novel surface-enhanced spectroscopies and plasmon lasers.

## Introduction

Since the pioneering work of Bender *et al*.^1^, who showed that the complex PT-symmetric potentials can have a real spectrum, significant attention has been devoted to quantum Hamiltonian systems with PT-symmetry^2–4^. Beyond some non-Hermiticity threshold, typically called the exceptional point, PT-symmetric systems can display an abrupt phase transition and the corresponding eigenspectra become complex. Recently, the concept of PT-symmetry has been fruitfully extended to wave optics. Photonic platforms are ideally suited to implement the structures with PT-symmetry, which is directly translated into a requirement for the arrangement of elements with balanced gain and loss^5–25^. Various intriguing optical phenomena have been revealed in PT-symmetric systems, such as asymmetric light propagation^7–9^ and invisibility^10, 11^, Bloch oscillation of energy^12^, coherent perfect laser absorber^13–17^, PT-symmetric metasurfaces^18–21^, single-mode laser^22, 23^, reversing the pump dependence of a laser^24^ and loss-induced suppression and revival of lasing^25^.

More recently, PT-symmetric systems built using plasmonic elements have attracted increased attention. The strong interaction between surface plasmons and electromagnetic field can enhance the extraordinary properties associated with exceptional points^26–30^. Besides the exceptional point, the spectral singularity^31, 32^ existing in the PT-plasmonic system, which is related to scattering resonance of the non-Hermitian Hamiltonian and manifests itself as giant transmission and reflection with vanishing bandwidth, also has unique features. For example, hugely anisotropic optical scattering^33^, three-dimensional light confinement^34^, and loss-induced super scattering^35, 36^.

On the other hand, there is a rapid progress in the field of graphene plasmonics motivated by the unique electrical and optical properties of graphene^37, 38^. For example, nanopatterned graphene can be used as an active medium for infrared electro-optic devices^39, 40^. Meanwhile, loss induced amplification of graphene plasmons^41^, regulation of energy distribution in graphene^42^, and singularity-enhanced sensing based on the PT-graphene metasurface^43^, as characteristics of exceptional point behaviors, have been demonstrated theoretically. However, characters of spectral singularities in graphene-based PT systems has not been discussed.

In this work, we systematically investigate characteristics of the eigenmode at spectral singularity when the electromagnetic field, in terahertz (THz) regime, is incident on an optically pumped monolayer graphene underneath the one-dimensional gain-loss diffractive grating. The intrinsic loss of graphene can exhibit negative, zero, and positive values resulting from the population inversion produced by cascaded optical-photon emission^44–47^. Such diverse surface dynamic conductivity responses can be used to manipulate optical modes of spectral singularities in graphene-based PT-systems. When the grating possesses perfect PT-symmetry, the spectral singularity will present the feature of lasing-mode (electric fields concentrate mainly on the gain element) or absorption-mode (electric fields concentrate mainly on the loss element) with the loss of optically pumped graphene being negative or positive, respectively. It is noted that spectral singularities are vanished if the loss of optically pumped graphene becomes zero. In particular, the spectral singularity may exhibit symmetry-modes (electric fields concentrate equally on loss and gain elements), when the loss and gain of the grating is unbalanced. In this case, the spectral singularity on the lasing or absorption-mode also appears with the loss and gain for the grating exceeding the value of the corresponding symmetry-modes.

## Results and Discussions

### Optically pumped graphene and graphene-based PT system

**Figure 1:**
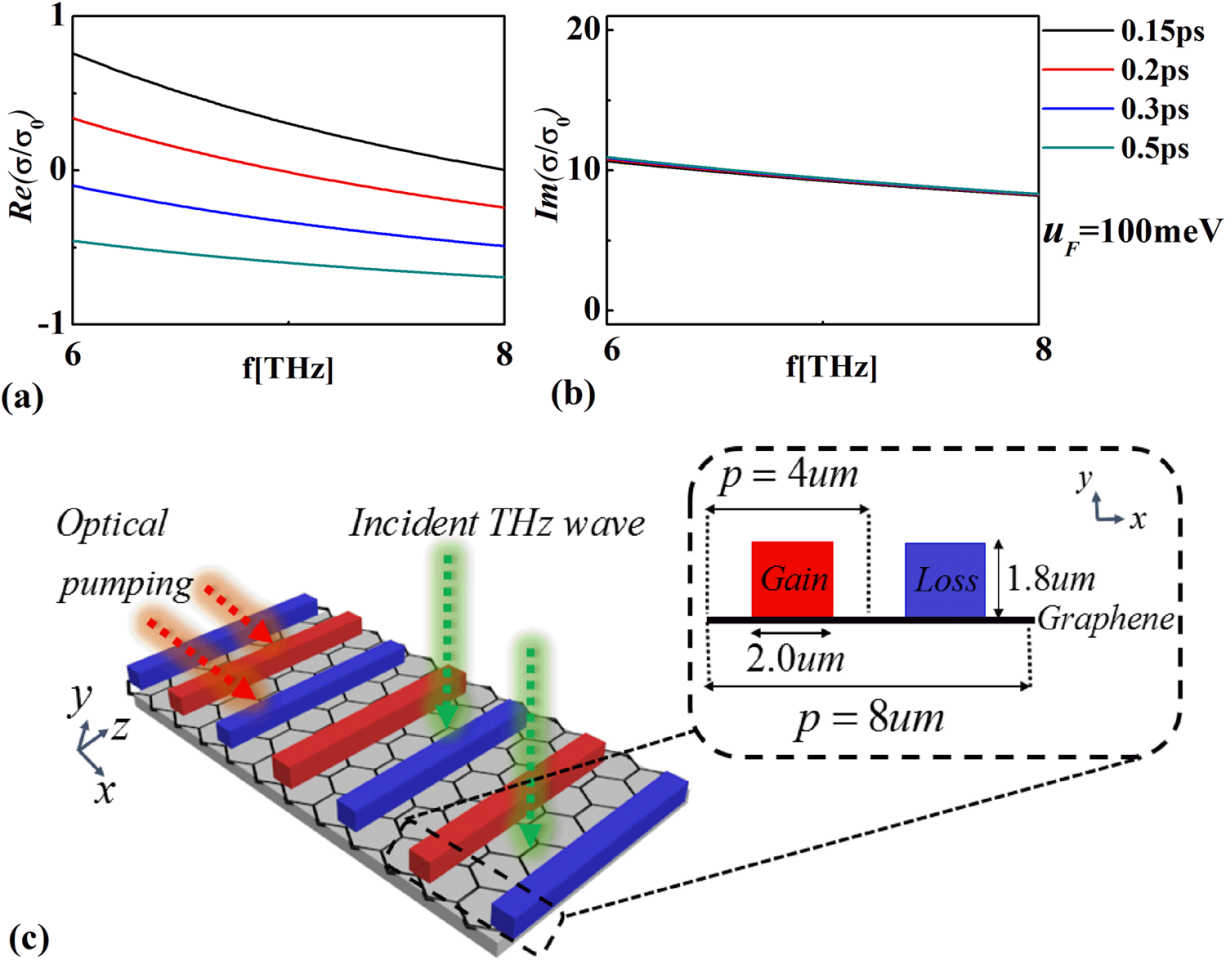
Real (**a**) and imaginary (**b**) parts of the conductivity of graphene as functions of the incident frequency with different *τ*. (**c**) The schematic of graphene-based system.

The nonequilibrium THz properties of graphene are especially interesting due to the population inversion and negative dynamic conductivity. The optical generation of electron-hole pairs in graphene can be described by quasi-Fermi-levels for electrons *u*_*Fe*_ and holes *u*_*Fh*_ of the same absolute value *u*_*Fe*_ = *−u*_*Fh*_ = *u*_*F*_. Since the relaxation time for intraband transition is much faster than the recombination time for electron-hole pairs, the population inversion can be achieved with optical pumping. In this condition, the complex intraband and interband conductivities, ${\sigma }_{intra}$ and ${\sigma }_{inter}$, can be approximately expressed as (in THz frequency)^48^:

1 $$\begin{array}{c}{\sigma }_{intra}=\frac{2{e}^{2}{k}_{B}T\tau }{\pi {\hslash }^{2}(1+{\omega }^{2}{\tau }^{2})}\,\mathrm{log}(1+{e}^{{\mu }_{F}/{k}_{B}T})+i\frac{2{e}^{2}{k}_{B}T\omega }{\pi {\hslash }^{2}({\omega }^{2}+1/{\tau }^{2})}\,\mathrm{log}(1+{e}^{{\mu }_{F}/{k}_{B}T})\\ {\sigma }_{inter}=\frac{{e}^{2}}{4{\hslash }^{2}}\,\tanh (\frac{\hslash \omega -2{\mu }_{F}}{4{k}_{B}T})+i\frac{{e}^{2}}{8\hslash \pi }\,\mathrm{log}[\frac{{(\hslash \omega +2{\mu }_{F})}^{2}}{{\hslash }^{2}{\omega }^{2}+4{{k}_{B}}^{2}{T}^{2}}],\end{array}$$

where $\omega$ is the angular frequency, *e* is the electric charge, $\hslash$ is the reduced Planck’s constant, ${k}_{B}$ is the Boltzmann constant, *T* is the temperature, and $\tau$ is the momentum relaxation time of charge carriers. In Fig. 1(a) and (b), we plot real and imaginary parts of the conductivity of graphene ($\sigma /{\sigma }_{0}$) as functions of the incident frequency with different $\tau$, respectively. Here, ${\sigma }_{0}$ equals to ${e}^{2}/4\hslash$ and $\sigma ={\sigma }_{intra}+{\sigma }_{inter}$. The temperature is *T* = 3 K and quasi-Fermi-level is *u*_*F*_ = 100 meV. As can be seen, the loss of graphene $Re(\sigma /{\sigma }_{0})$ can be continuously tuned from negative to positive by just varying the value of $\tau$. In this condition, the imaginary part of conductivity of graphene is nearly invariable. The frequency range considered here is starting from 6 THz to 8 THz, which is consistent with the operating wavelength for the designed system associated with photoexcited graphene. The corresponding schematic diagram is shown in Fig. 1(c). Here, we use an optically pumped graphene underneath the one-dimensional gain-loss diffractive grating (infinite along z-axis) to facilitate its plasmon excitation^49^. The amplifying view of a unit cell is clearly presented in the inset of Fig. 1(c). The gain-loss elements repeat in x-axis with the period being *p* = 8 um. The sizes of the grating along x- and y-axis are 2.0 um and 1.8 um, respectively. The relative permittivities of the gratings are given by ${\varepsilon }_{gain}=10(1-j{F}_{gain})$ and ${\varepsilon }_{loss}=10(1+j{F}_{loss})$ with *F*_*gain*_ and *F*_*loss*_ being the non-Hermiticity coefficients of the system. It is noted that the whole structure possesses the PT-symmetry, only when distributions of gain/loss elements are balanced (*F*_*gain*_ = *F*_*loss*_) and the dynamic conductivity of graphene becomes zero ($Re(\sigma /{\sigma }_{0})=0$).

### PT-diffractive grating and lossless monolayer grapheme

**Figure 2:**
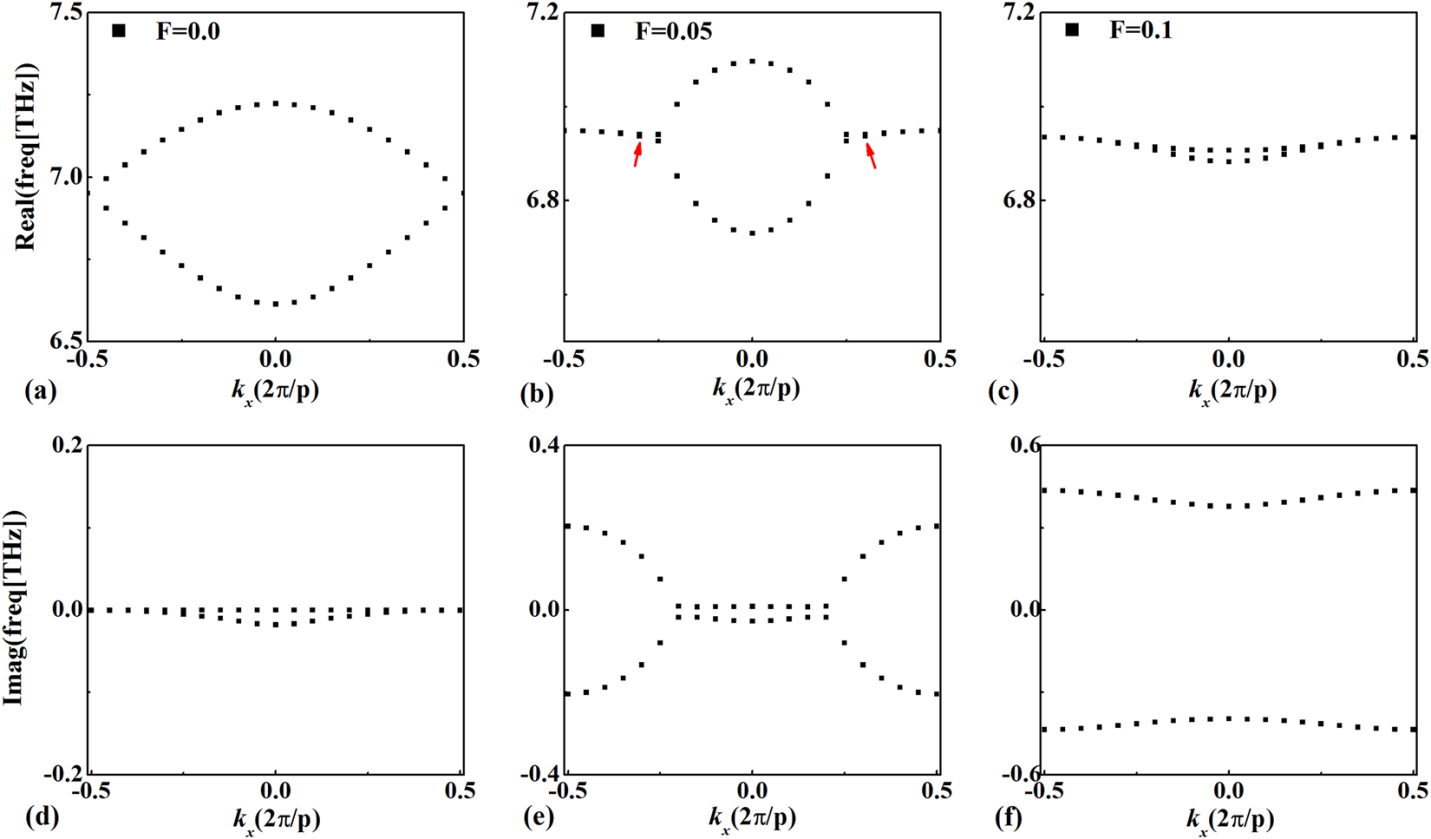
Complex band structures with non-Hermiticity coefficient *F* being 0.0 (**a**), (**d**), 0.05 (**b**), (**e**) and 0.1 (**c**), (**f**). The loss of graphene is zero.

Firstly, we proceed to investigate the interaction between the PT-diffraction grating and lossless monolayer graphene ($Re[\sigma /{\sigma }_{0}]=0.0$), where the whole structure is PT-symmetric. The dispersion relations between the eigenfrequency and Bloch wave vector can be calculated by using finite element method (Comsol Multiphysics 5.2a). In Fig. 2(a) and (d), we plot the complex dispersion curves with non-Hermiticity coefficient being zero (*F*_*gain*_ = *F*_*loss*_ = 0). Only two modes are considered here with eigenfrequencies (real part) locating within 6–8 THz. Although the system has no loss or gain element, the imaginary part of eigenfrequency is still non-zero resulting from the existence of radiation loss, which is largest at the Brillouin center and vanished at Brillouin boundaries. As we turn on the gain and loss (*F*_*gain*_ = *F*_*loss*_ = 0.05), the gap of the real part of eigenfrequency at the Brillouin boundary will be closed and the imaginary part separated, giving rise to the exception point (red arrow), as shown in Fig. 2(b) and (e). Due to the non-ignorable radiation loss existing at the Brillouin center, Fig. 2(d) shows that the real part of eigenfrequency cannot coalesce fully even in the PT-broken phase. However, a pair of lasing- and absorption-modes are still formed with the imaginary parts of eigenfrequency completely separated, as shown in Fig. 2(f).

**Figure 3:**
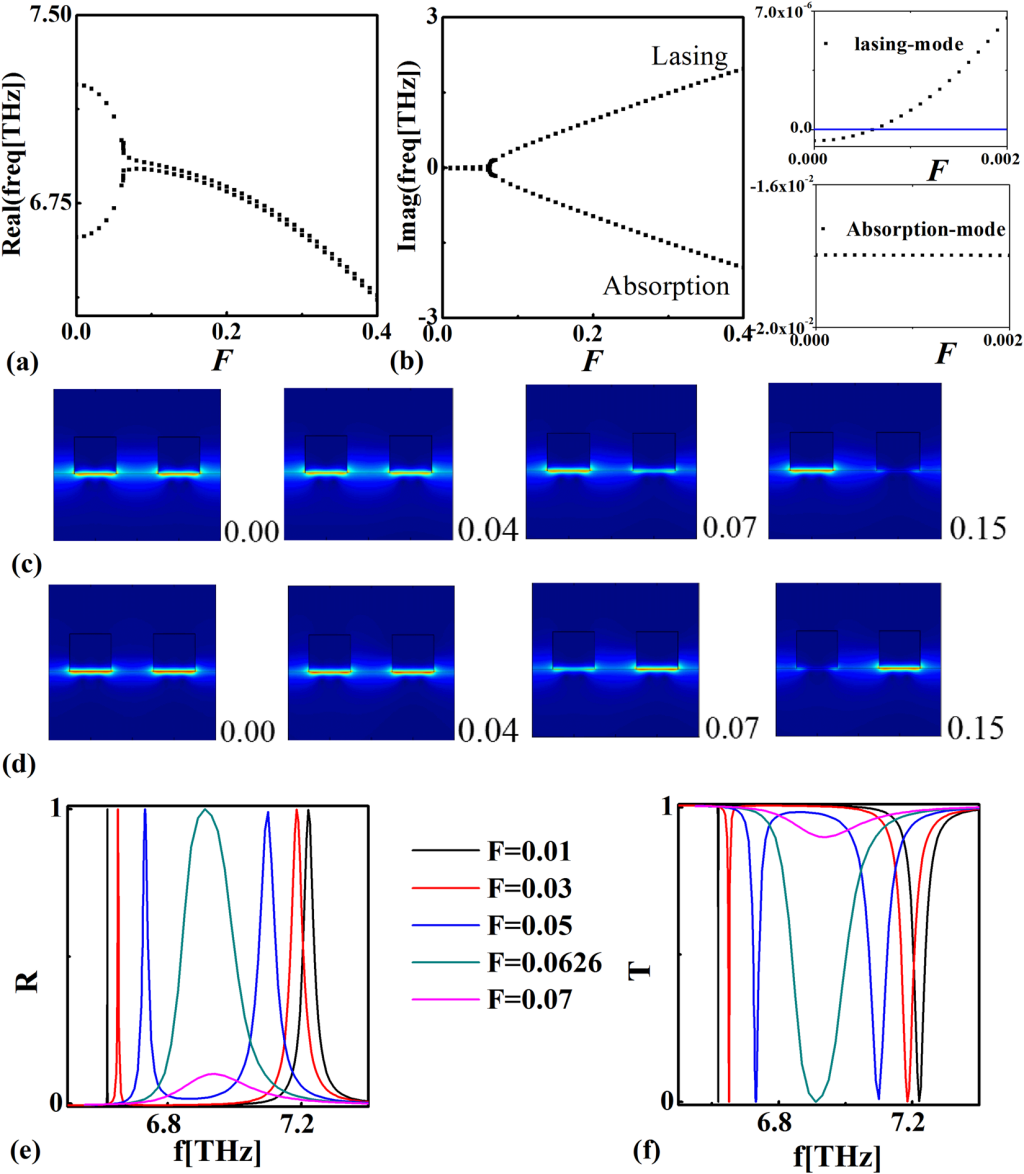
Real (**a**) and imaginary (**b**) parts of eigenfrequencies (*k*_*x*_ = 0) as functions of *F* with the loss of graphene being zero. The inset of Fig. 3(b) shows the imaginary parts of eigenfrequencies as functions of the non-Hermiticity coefficient *F* (ranging from 0.0*-*0.002). (**c**) and (**d**) show evolutions of the electric field distribution of the lasing- and absorption-modes by varying the non-Hermiticity coefficient. (**e**) and (**f**) show the reflectance and transmittance of the PT*-*system with different values of the non-Hermiticity coefficient.

In order to observe the evolution of eigenmode with the variation of non-Hermiticity coefficient, in Fig. 3(a) and (b), we plot real and imaginary parts of eigenfrequencies at Brillouin center as functions of the non-Hermiticity coefficient *F*_*gain*_ = *F*_*loss*_ = *F*, respectively. We find that real parts of eigenfrequencies nearly coalesced and imaginary parts completely separated, when the parameter *F* increases to a critical value about 0.0626. In this condition, the PT symmetry is broken. It is worthy to note that imaginary parts of eigenfrequencies have already separated in a small degree before *F* reaches to the critical value (~0.0626), as shown in the inset of Fig. 3(b). This phenomenon stems from the existence of radiation loss in open PT-symmetric systems. On the other hand, comparing with the absorption loss, the radiation loss is negligible. Consequently, before the breaking threshold (F~0.0626) is reached, the electric fields are nearly symmetrically distributed on the loss and gain elements and no spectral singularity appears. Figure 3(c) and (d) present evolutions of lasing- and absorption-modes fields with the non-Hermiticity coefficient (shown in lower right corners) being varied. When the PT-symmetry is broken, the electric field is confined mainly in the amplification section for the lasing-mode, whereas the absorption-mode is loss-dominant with electric field mainly concentrated on the loss section. To investigate the scattering property of this PT-system, we plot the reflectance and transmittance as functions of the incident frequency with different non-Hermiticity coefficients *F*, as shown in Fig. 3(e) and (f). We find that two peaks (valleys) of the reflectance (transmittance) gradually approach to each other with increasing non-Hermiticity coefficients, and merge into one peak at the exceptional point. If we further increase the non-Hermiticity coefficients, the reflectance (transmittance) peaks (valleys) gradually disappeared. The frequencies of reflectance peaks are consistent with real parts of eigenfrequencies calculated in Fig. 3(a). It is worthy to note that although significant gain and loss exist in this perfect PT-system, the sum of the transmittance and reflectance nearly equals to one, and no spectral singularity appears.

### PT-diffractive grating and passive or active monolayer grapheme

**Figure 4:**
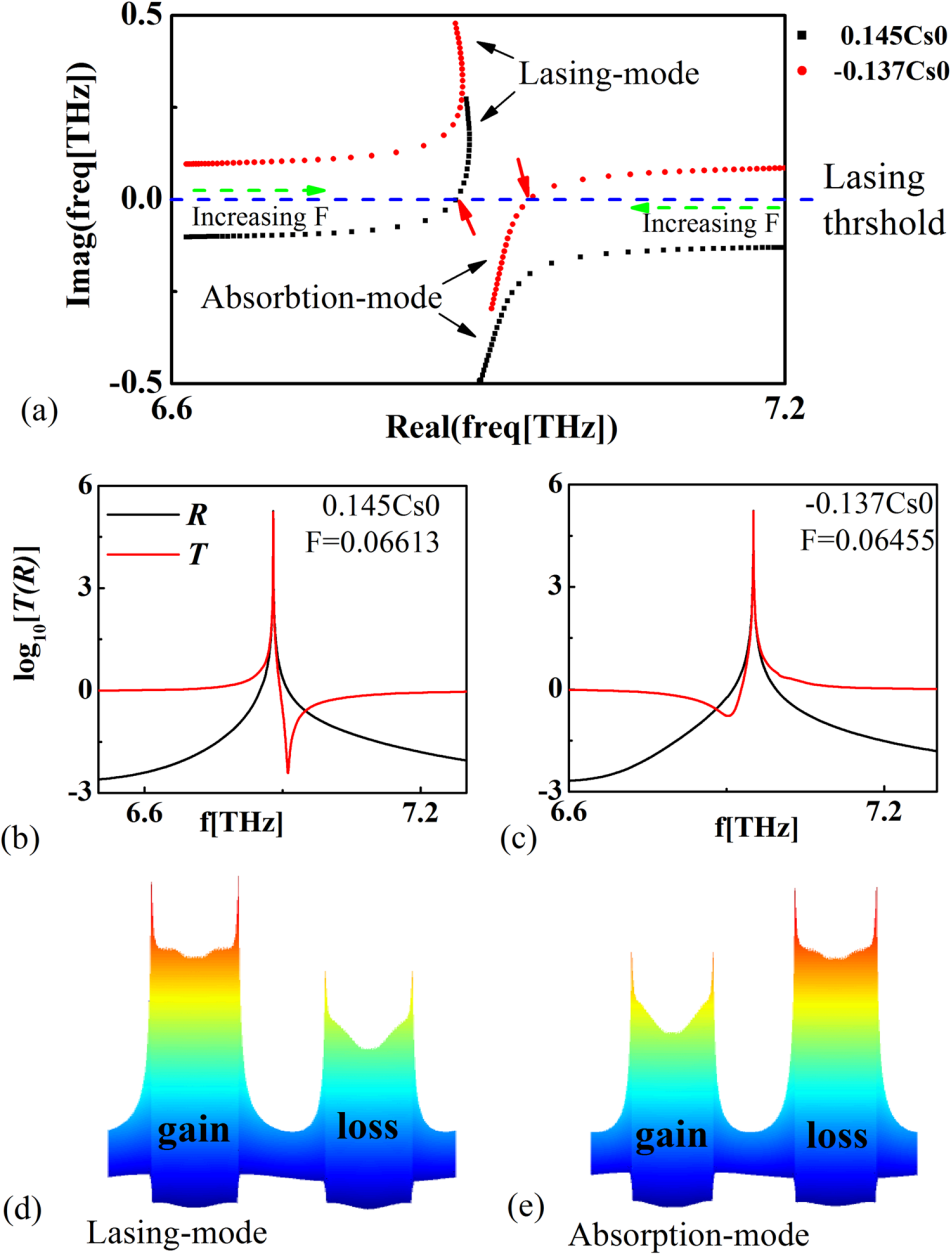
(**a**) Movement of the eigenfrequencies for the states at *k*_*x*_ = 0 in the complex plane with the surface dynamic conductively of graphene being $Re[\sigma /{\sigma }_{0}]=0.145$ (black dot line) and $Re[\sigma /{\sigma }_{0}]=-0.137$ (red dot line). (**b**) and (**c**) show the transmittance and reflectance of the normal incident wave with the surface dynamic conductivity of graphene being negative (*F* = 0.06613) and positive (*F* = 0.06455), respectively. (**d**) and (**e**) show the corresponding near-field distributions at spectral singularities.

The property of the system changes dramatically when the loss of graphene becomes non-zero (the whole structure is not PT-symmetric). The evolutions of eigenfrequencies for the states at *k*_*x*_ = 0 in a complex frequency plane are shown in Fig. 4(a). The green dash arrows point the evolution directions of eigenfrequency with *F* being increased. The black dot line represents the condition that the real part of the surface conductivity of graphene is positive ($Re[\sigma /{\sigma }_{0}]=0.145$, passive). In this case, the eigenfrequency on the lasing-mode approaches to the lasing threshold (blue dash line). While, the absorption-mode leaves away from it. Thus, the spectral singularity (marked by the red arrow) appears on the lasing-mode. When we change the real part of the surface conductivity of graphene to be negative ($Re[\sigma /{\sigma }_{0}]=-0.137$, active, red dot line), the corresponding spectral singularity appears on the absorption-mode. Consequently, the spectral singularity can be tuned from lasing-mode to absorption-mode by just changing the surface conductivity of graphene from passive to active. It is extremely different from the PT-plasmonic systems, where the spectral singularity only exists on the lasing-mode due to the metallic materials are uniformly lossy^34–36^.

The spectral singularity manifests itself as giant transmission and reflection with vanishing bandwidth. In Fig. 4(b) and (c), we plot the transmittance (red line) and reflectance (black line) at spectral singularities with the surface dynamic conductivity of graphene being negative and positive, respectively. The corresponding non-Hermiticity coefficients are *F* = 0.06613 and *F* = 0.06455, respectively. It is presented that the giant transmittance and reflectance appear, and corresponding near-field distributions are shown in Fig. 4(d) and (e). When the surface resistance of graphene is positive (passive), the electric field is mainly confined on the amplification sections (corresponding to the lasing-mode). While, the electric field is concentrated on the loss element mostly (corresponding to the absorption-mode), when the surface resistance of graphene is negative (active). Also, the frequencies correspond to the giant transmittance and reflectance are consistent with the eigenfrequencies calculated in Fig. 4(a). In contrast to the previous method to control the near-field by modifying the geometrical parameters of the plasmonic structures or the surrounding dielectric environment, we can tune the near-field distributions by just varying the surface dynamic conductivity of graphene.

### Non*-*PT*-*diffractive grating and passive or active monolayer grapheme

**Figure 5:**
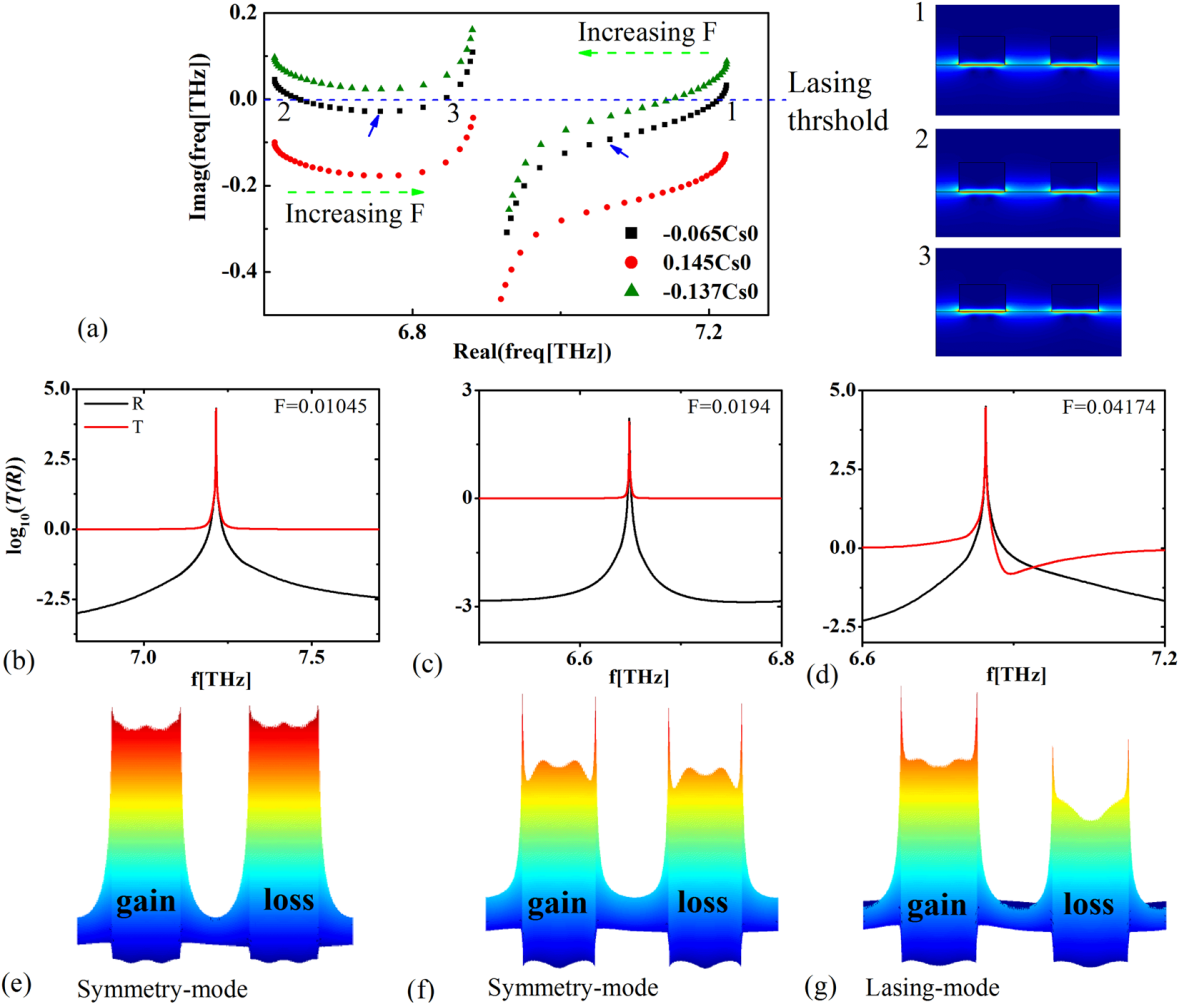
Non-PT grating 2*F*_*gain*_ = *F*_*loss*_ = *F*. (**a**) Movement of the eigenfrequencies for the states at *k*_*x*_ = 0 in the complex plane when the surface dynamic conductively of graphene being $\mathrm{Re}[\sigma /{\sigma }_{0}]=-0.065$ (black dot line), $\mathrm{Re}[\sigma /{\sigma }_{0}]=0.145$(red dot line), and $\mathrm{Re}[\sigma /{\sigma }_{0}]=-0.137$ (green dot line). The right insets show the eigenmode fields at three (1, 2, 3) spectral singularities. (**b**)–(**d**) show the transmittance and reflectance of the normal incident wave on the graphene-based quasi-PT system with the surface dynamic conductivity of graphene being $\mathrm{Re}[\sigma /{\sigma }_{0}]=-0.065$ and the non-Hermiticity coefficients being *F* = 0.01045, *F* = 0.0194 and *F* = 0.04174, respectively. (**e**)–(**g**) show the near-field distributions at three spectral singularities (1, 2, 3).

Finally, we will investigate the interplay between a non-PT diffraction grating, 2*F*_*gain*_ = *F*_*loss*_ = *F*, and the optically pumped graphene. The evolutions of the eigenfrequencies for the states at *k*_*x*_ = 0 in a complex frequency plane are shown in Fig. 5(a). The green dash arrows point the directions with *F* being increased. When the surface dynamic conductivity of graphene is active $Re[\sigma /{\sigma }_{0}]=-0.065$ (the black dot line), three spectral singularities exist (marked by **1, 2, 3**) and each possesses different eigenmodes, shown in right insets of Fig. 5(a). Two of them (**1, 2**) possess symmetric eigenmodes before the eigenfrequency passes through the inflection points (marked by the blue arrows). Beyond the inflection points, the lasing-mode, which previously left away from the lasing threshold, will approach to it again and the corresponding spectral singularity reappears. This phenomenon follows the main characteristics of the quasi-PT-systems with exceptional points, like loss-induced suppression and revival of lasing^15^, and reversing the pump dependence of a laser^14^. It is noted that spectral singularities only exhibit symmetry-modes, when the distributions of the loss and gain for the diffractive grating are unbalanced. Moreover, in Fig. 5(b–d), we plot the reflectance (black line) and transmittance (red line) at these three spectral singularities. Reasonably, the giant transmission and reflection happened. The corresponding near-field distributions are plot in Fig. 5(e,f), which are consistent with eigenmode fields. In addition, if we change the value of surface dynamic conductivity of graphene, the number of the spectral singularity many be reduced. Only symmetric mode exists with the surface dynamic conductivity of graphene being $Re[\sigma /{\sigma }_{0}]=-0.137$ (green dot line in Fig. 5(a)), and the lasing-mode exists with the surface dynamic conductivity of graphene being $Re[\sigma /{\sigma }_{0}]=0.145$ (red dot line in Fig. 5(a)).

**Figure 6:**
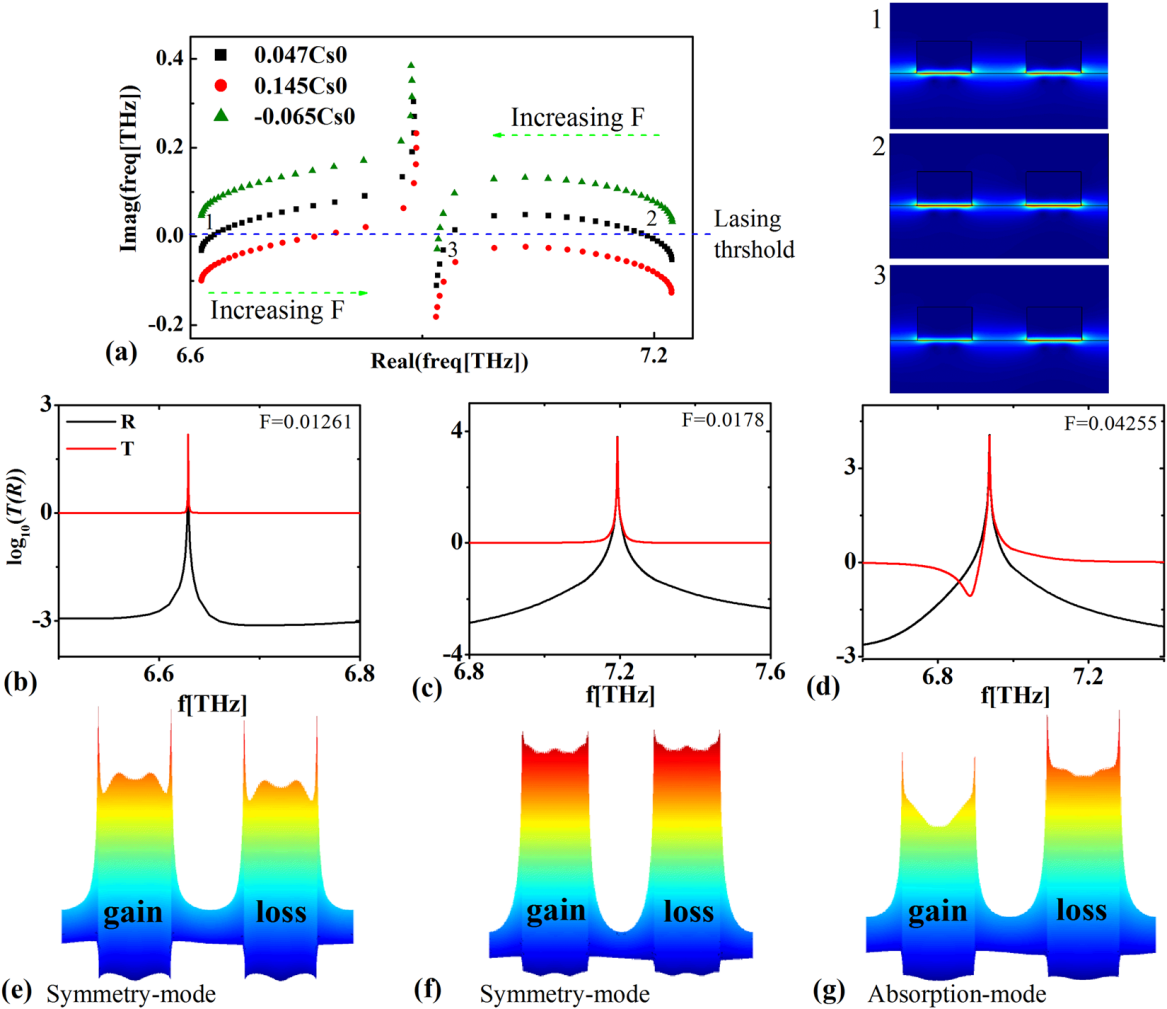
Non-PT grating *F*_*gain*_ = 2*F*_*loss*_ *=* *F*. (**a**) Movement of the eigenfrequencies for the states at *k*_*x*_ = 0 in the complex plane when the surface dynamic conductively of graphene being $\mathrm{Re}[\sigma /{\sigma }_{0}]=0.047$ (black dot line), $\mathrm{Re}[\sigma /{\sigma }_{0}]=0.145$ (red dot line), and $\mathrm{Re}[\sigma /{\sigma }_{0}]=-0.065$ (green dot line). The right insets show the electric field distributions at three (1, 2, 3) spectral singularities. (**b**–**d**) show the transmittance and reflectance of the normal incident wave on the graphene-based quasi-PT system with the surface dynamic conductivity of graphene being $\mathrm{Re}[\sigma /{\sigma }_{0}]=0.047$ and the non-Hermiticity coefficients being *F* = 0.01261, *F* = 0.0178 and *F* = 0.04255, respectively. (**e**–**g**) show the near-field distributions at three spectral singularities (1, 2, 3).

We also consider the condition, when non-Hermiticity coefficients satisfied the relationship of *F*_*gain*_ = 2*F*_*loss*_ = *F*. Characteristics of the corresponding spectral singularities in a manner analogous to the above case are shown in Fig. 6. When the surface dynamic conductivity of graphene is passive $Re[\sigma /{\sigma }_{0}]=0.047$, three spectral singularities exist. Two of them (**1, 2**) possess symmetric eigenmodes and another one presents features of the absorption-mode. Similarly, the giant transmission and reflection are also excited at frequencies of the corresponding spectral singularities.

## Conclusions

In conclusion, we have demonstrated numerically that eigenmodes at spectral singularities can be conveniently tuned by a suitable variation of the loss and gain in the graphene-based quasi-PT systems. When the diffractive grating, which assisted graphene plasmonics excitation, has the perfect PT-symmetry, the spectral singularity can present the feature of lasing- or absorption-modes, which is decided by the intrinsic property of loss or gain characteristic for the surface conductivity of the pumped graphene. These spectral singularities vanished if the surface resistance of graphene becomes zero. In particular, the spectral singularity may exhibit symmetry-mode only with the asymmetric distribution of the loss and gain for the diffractive grating. Furthermore, with the increasing of non-Hermiticity coefficients, the spectral singularities with asymmetric eigenmodes reappeared. In contrast to the previous method to control the near-field by modifying the geometrical parameters of the plasmonic structures, we can tune the near-field distributions around the graphene-based PT-system by just varying the surface dynamic conductivity of graphene. We hope that our finding may have some applications in designing novel surface-enhanced spectroscopies and plasmon lasers.

## Methods

All full wave numerical simulations and dispersion relations were done using finite element method (Comsol Multiphysics). In the simulations, the graphene is modeled as a two-dimensional surface with complex conductivity. The mesh size inside the graphene layer is 0.5 nm, which is fine enough for convergence.
